# Aligning Out-of-Plane Polarization in 2D Ferroelectric Perovskite Wafer Boosts Interlayer Carrier Transport for Efficient X-Ray Detection

**DOI:** 10.1007/s40820-026-02352-z

**Published:** 2026-10-15

**Authors:** Chenhao Deng, Shihai You, Haiqing Zhong, Xiankan Zeng, Xiaolong Hao, Jiasheng Xie, Zheng Zhou, Zhangjian Jin, Huang Ye, Lijun Xu, Jianbo Wu, Panpan Dong, Wen Li, Xingxing Jiao, Weiqing Yang

**Affiliations:** 1 Key Laboratory of Advanced Technologies of Materials (Ministry of Education), School of Materials Science and Engineering, Research Institute of Frontier Science, Southwest Jiaotong University, Chengdu, 610031 People’s Republic of China; 2 State Key Laboratory of Functional Crystals and Devices, Fujian Institute of Research on the Structure of Matter, Chinese Academy of Sciences, Fuzhou, 350002 People’s Republic of China; 3 Department of Materials Science and Engineering, City University of Hong Kong, Hong Kong SAR, 999077 People’s Republic of China

**Keywords:** 2D perovskite, X-ray detection, Polycrystalline wafer, Hot-pressing, Out-of-plane polarization

## Abstract

2D metal halide perovskites (MHPs) featuring suppressed ion migration and enhanced structural stability are highly promising for direct X-ray detection but suffer from poor interlayer carrier transport and difficulty in scaling single crystals. Here, we overcome these limitations by fabricating high-quality, centimeter-sized polycrystalline wafers of a 2D ferroelectric MHP with out-of-plane spontaneous polarization, (Mpda)PbBr_4_ (Mpda = *N*-methyl-1,3-propanediamine), via a facile hot-pressing strategy. This process induces a horizontal orientation of microcrystals, resulting in vertical alignment of their polar axes and thus macroscopic polarization in the wafer. Such an intriguing polarization generates a switchable built-in electric field that can promote separation and interlayer transport of photogenerated carriers, exhibiting a bulk photovoltage of up to 2 V. Consequently, the vertical X-ray detector can not only be operated in self-powered mode but also achieve a high sensitivity of 2285 *μ*C Gy^−1^ cm^−2^ and an ultralow detection limit of 7.2 nGy s^−1^ under a small bias (16 V). The device also exhibits remarkable operational stability and high-resolution X-ray imaging capability. This work demonstrates that aligning the intrinsic ferroelectric polarization in polycrystalline wafers is a viable strategy to enhance vertical carrier transport, paving the way toward high-performance, flat-panel X-ray detectors based on 2D MHPs.

**Supplementary Information:**

The online version contains supplementary material available at https://doi.org/10.1007/s40820-026-02352-z.

## Hightlights

- High-quality, centimeter-sized polycrystalline wafers of a 2D ferroelectric perovskite are fabricated via a facile hot-pressing method, circumventing the challenge of growing large, thick single crystals of 2D perovskites.
- The preferred horizontal orientation of grains, combined with electric poling, creates macroscopic out-of-plane polarization, generating a strong built-in electric field that boosts interlayer carrier transport.
- The poled wafer not only enables self-powered X-ray detection but also achieves a high sensitivity (2285 μC Gy^−1^ cm^−2^) and an ultralow detection limit (7.2 nGy s^−1^) under a small bias, along with exceptional operational stability and high-contrast X-ray imaging.

**Supplementary Information:**

The online version contains supplementary material available at https://doi.org/10.1007/s40820-026-02352-z.

## Graphical abstract

**Figure:**
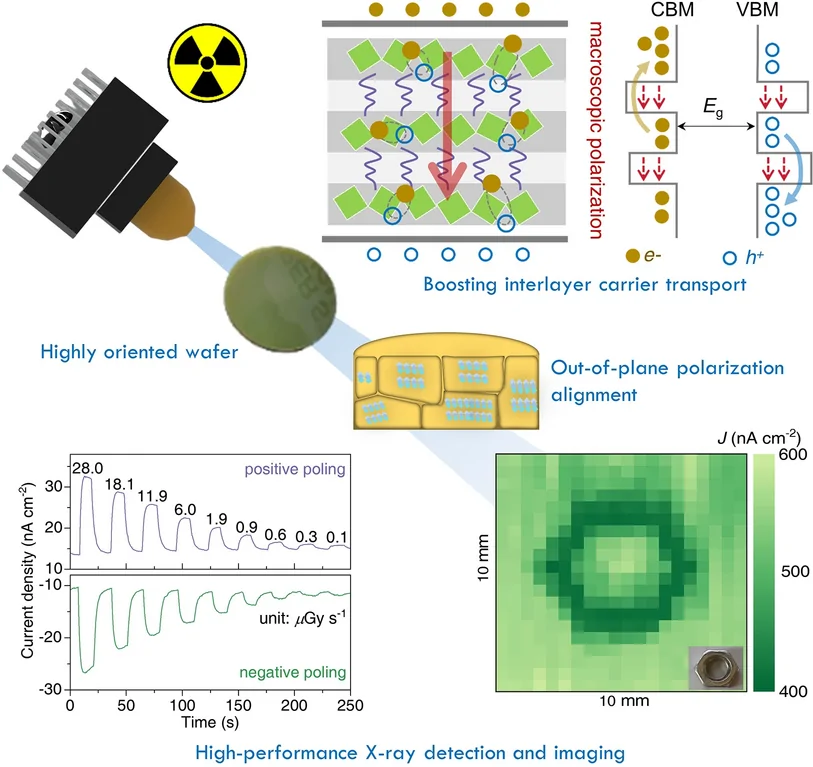

## Introduction

Metal halide perovskites (MHPs) have emerged as a star class of optoelectronic semiconductors for direct X-ray detection, owing to their strong X-ray attenuation, high carrier mobility-lifetime product (*μτ*), and facile solution-processed synthesis [1–7]. Significant progress has been achieved in the past decade. For instance, a high sensitivity of 2.16×10^5^ *μ*C Gy^−1^ cm^−2^ and an ultralow detection limit of 2 nGy s^−1^ have been reported in a 3D FAPbI_3_ (FA = formamidine) single-crystal X-ray detector [8], far surpassing those of commercial amorphous selenium (*α*-Se) film detectors (~25 *μ*C Gy^−1^ cm^−2^ and 5500 nGy s^−1^) by several orders of magnitude. However, the practical deployment of 3D MHPs is hindered by intrinsic structural instability and severe ion migration, which compromises their long-term working stability [9–11]. In this context, 2D MHPs, naturally adopting a motif of alternating organic layers and inorganic slabs, offer enhanced environmental stability, suppressed ion migration, and reduced leakage currents, making them an ideal subclass for direct X-ray detection [12–16]. Recently, a series of 2D MHPs including Ruddlesden–Popper (RP) and Dion–Jacobson (DJ) phases, such as (F-PEA)_2_PbI_4_ (F-PEA = fluorophenethylammonium) and (BDA)PbI_4_ (BDA = 1,4-butanediamine), have been developed and indeed exhibited superior working stability in direct X-ray detectors [17–25]. However, the pronounced dielectric contrast between the organic spacer and the inorganic metal halide layers creates a multi-quantum-well electronic structure in 2D MHPs, thus leading to strong quantum and dielectric confinement effects. This inherent property severely limits the efficient transport of carriers especially the interlayer (out-of-plane) transport between inorganic layers [12, 26–28], resulting in generally inferior detection sensitivity than their 3D counterparts. Therefore, enhancing interlayer carrier transport in 2D MHPs is crucial for advancing their performance in practical X-ray detection applications.

In recent years, the intrinsic spontaneous polarization in ferroelectric or polar MHPs has attracted considerable interest in optoelectronics [29–31]. This polarization generates a built-in electric field that drives the spontaneous separation and transport of photogenerated electrons and holes, leading to a stable bulk photovoltaic effect (BPVE) under light irradiation [32–35]. Leveraging this effect has significantly improved the performance of various MHP-based optoelectronic devices, including solar cells and photodetectors [26, 36]. For instance, Wang et al. utilized the 2D ferroelectric MHP (DFPD)_2_PbI_4_ (DFPD = 4,4-difluoropiperidinium) as the absorbing layer in a film solar cell, where the polarization-induced built-in electric field enhanced out-of-plane carrier transport, breaking the limit of multi-quantum-well electronic structure and achieving a high efficiency of 3.71% among 2D RP MHPs [26]. Similarly, Luo’s group developed a series of polar MHPs whose spontaneous polarization enabled pronounced BPVE under X-ray irradiation, facilitating the creation of high-performance, self-powered single-crystal X-ray detectors [23, 30, 32, 37–41]. These works reveal that introducing polarization is a promising strategy to boost the performance of 2D MHP-based X-ray detectors.

However, two major challenges impede the application of this strategy in practical X-ray detection. First, in most reported 2D polar MHPs, the polarization axis lies parallel to the inorganic layers (e.g., in-plane polarization) [42, 43]. This does not enhance the critical interlayer (out-of-plane) carrier transport, rendering it incompatible with the standard vertical architecture of commercial X-ray detectors. Second, practical devices require X-ray detection materials with scalable area and sufficient thickness for effective X-ray attenuation and imaging. Unfortunately, the growth of large, thick single crystals of 2D MHPs remains challenging and time-consuming, primarily owing to the complex molecular configuration of large organic cations which hinders ordered crystal growth along the vertical direction [44–46]. In this context, fabricating large-area 2D MHPs that simultaneously possess out-of-plane polarization is of great significance for advancing high-performance X-ray detection.

In this work, we address these challenges through fabricating centimeter-sized polycrystalline wafers of the 2D ferroelectric MHP, (Mpda)PbBr_4_ (Mpda = *N*-methyl-1,3-propanediamine), via a facile hot-pressing method. This process induces a horizontal orientation of micrograins, enabling the vertical alignment of their intrinsic polarization axes and resulting in macroscopic out-of-plane polarization across the entire wafer. This unified polarization creates a robust built-in electric field that efficiently drives the separation and interlayer transport of photogenerated carriers, exhibiting a strong, switchable BPVE (~ 2 V) under X-ray irradiation. Constructing vertical X-ray detectors based on high-quality (Mpda)PbBr_4_ wafers achieves a high sensitivity of 2285 *μ*C Gy^−1^ cm^−2^ and an ultralow detection limit of 7.2 nGy s^−1^ even under a small external bias. Moreover, this device also demonstrates excellent operational stability as well as high-resolution imaging capability. This study successfully integrates inherent out-of-plane polarization into scalable 2D MHP polycrystalline wafers, providing new insights into the design and fabrication of high-performance X-ray detectors.

## Experimental Section

### 2.1 Materials

Starting materials, lead acetate trihydrate (Pb(CH_3_COO)_2_·3H_2_O, 99.5%, Aladdin), hydrobromic acid (HBr, 48 wt% in water, Aladdin), *N*-methyl-1,3-propanediamine (Mpda, 98%, Aladdin), are commercially available and used without further purification.

### Preparation and Characterization

#### ***Synthesis of (Mpda)PbBr***_***4***_*** and Wafer Fabrication***

(Mpda)PbBr_4_ microcrystals were synthesized by dissolving Pb(CH_3_COO)_2_·3H_2_O (1 mmol, 379 mg) and Mpda (1 mmol, 88 mg) into 5 mL HBr solution through heating (100 °C) and constant magnetic stirring. After a clear solution was obtained, stop heating and stirring, and the mixture solution was left on the hotplate to cool to room temperature. Finally, light-yellow microcrystals were precipitated.

The microcrystalline powders (e.g., 0.5 g) of (Mpda)PbBr_4_ were ground for 20 min to ensure uniformity and then were transferred into a stainless-steel hot-pressing mold. First, the mold was heated to the setting temperature (e.g., 170 °C) and preheated for 10 min to soften these powders. Uniaxial pressure was applied at a rate of 30 MPa min^−1^ to the setting pressure (e.g., 300 MPa) and held for 60 min under continuous heating. The pressure was manually adjusted during hot-pressing to maintain the fluctuation within 10 MPa and ensure stability. After cooling to room temperature, the pressure was released manually and a high-quality wafer was demolded.

#### Characterization

The X-ray diffraction (XRD) patterns were acquired on a powder X-ray diffractometer (Empyrean, Malvern Panalytical) with Cu K*α*1 radiation (*λ* = 0.154 nm). Second harmonic generation (SHG) spectra were carried out on polycrystals using a Nd:YAG laser with the wavelength of 1064 nm, pulse duration of 5 ns, peak power of ~ 1.6 MW, and repetition rate of 10 Hz. The thermogravimetry (TG) curve was measured on a simultaneous thermal analyzer (STA 449 F3 Jupiter, Netzsch) in a N_2_ atmosphere from room temperature to 700 °C with a temperature increasing rate of 10 °C min^−1^. The absorption spectra were recorded on an ultraviolet–visible (UV–*Vis*) spectrometer (Lambda 950, PerkinElmer) from 200 to 800 nm. The ultraviolet photoelectron spectroscopy (UPS) spectrum was performed on film using an ultraviolet photoelectron spectrometer (QXi, ECSALAB) with He I as the radiation source. X-ray photoelectron spectroscopy (XPS) spectrum was collected on an X-ray photoelectron spectrometer (K-Alpha, Thermo Scientific). Scanning electron microscope (SEM) images and corresponding energy-dispersive spectrometer (EDS) mappings were obtained by a field emission scanning electron microscope (JSM7800-F, JEOL) equipped with energy-dispersive X-ray spectroscope (X-Max 80, Oxford). The atomic force microscope (AFM) image was measured using an atomic force microscope (Dimension ICON, Bruker). The piezoresponse force microscopy (PFM) measurement of the spin-coated (Mpda)PbBr_4_ film was carried out using a resonant-enhanced piezoresponse force microscope (MFP-3D, Asylum Research) equipped with conductive Pt/Ir-coated silicon tips (EFM-50, Nanoworld). The film was coated on an indium tin oxide (ITO) substrate. The electrical properties, e.g., current–voltage (*I*-*V*) curve, resistivity, temperature-dependent conductivity, were tested using a high-precision electrometer (6517B, Keithley).

#### Device Fabrication and X-Ray Detection

The vertical X-ray detectors were fabricated by coating silver conductive paste (SPI) on the top and bottom sides of diced (Mpda)PbBr_4_ wafer, and the electrode area is about 1 mm^2^ and the wafer thickness is 0.6 mm. A commercial X-ray tube with tungsten target (Magpro TUB155, Moxtek) operated at 50 kV tube voltage was used as the X-ray source, and aluminum plate was used as the attenuator. The X-ray dose rate was tuned by changing its tube current and measured using an X-ray dosimeter (Accu-Gold, Radcal) with ion chamber (10×6−180 model). The *I*-*V*, voltage–time (*V*-*t*), and current–time (*I*-*t*) curves were recorded on the high-precision electrometer (6517B, Keithley). The X-ray imaging was carried out by placing the object (a metal nut) on an electric X-Y displacement stage and collecting the photocurrent at each position. The scanning was performed over an area of 10 mm × 10 mm with a step size of 0.5 mm, yielding a pixel matrix of 20 × 20 (400 pixels in total). The X-ray dose rate and bias voltage for imaging were 60 *µ*Gy s^−1^ and 20 V, respectively. The acquired photocurrent values were then mapped into a matrix to reconstruct the X-ray image.

### Computation

The density functional theory (DFT) calculations were performed on the CASTEP module in Materials Studio. The PBE function in the framework of generalized gradient approximation (GGA) was used to deal with the exchange–correlation effects. An energy cutoff of 830 eV was used, and the k-space integration was carried out with a Monkhorst–Pack grid of 2×1×1. The interactions between the ionic cores and electrons were described using the norm-conserving pseudopotential. The orbital electrons of C (2*s*^2^2*p*^2^), N (2*s*^2^2*p*^3^), H (1s^1^), Pb (5*d*^10^6*s*^2^6*p*^2^), and Br (4*s*^2^4*p*^5^) were regarded as valence electrons.

## Results and Discussion

### Crystal Structure and Semiconducting Property

**Fig. 1:**
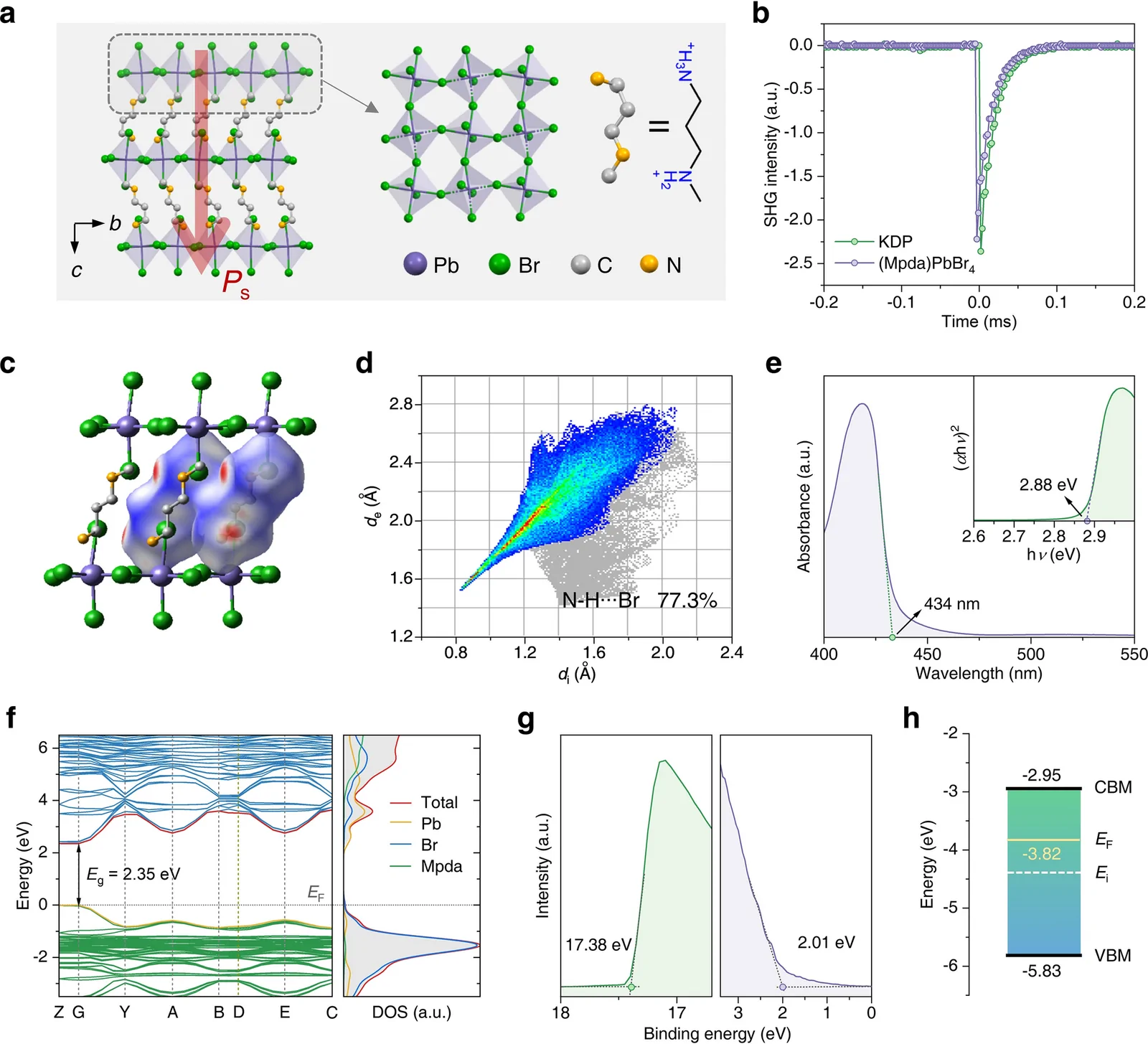
Crystal structure and semiconducting properties of (Mpda)PbBr_4_. **a** Crystal structure of (Mpda)PbBr_4_ viewed along the *a*-axis; the red arrow presents its spontaneous polarization along out-of-plane direction. **b** SHG signals of (Mpda)PbBr_4_ powders using KDP as a reference. **c, d** Hirshfeld surface of Mpda cation and corresponding 2D fingerprint maps for N-H···Br hydrogen bonds. **e** Absorption spectrum of (Mpda)PbBr_4_ powders, and the inset displays corresponding optical bandgap. **f** Calculated band structure and total and partial DOS profiles of (Mpda)PbBr_4_ using DFT method. **g, h** UPS spectrum and energy-level diagram of (Mpda)PbBr_4_

(Mpda)PbBr_4_ is a 2D perovskite ferroelectric induced by the temperature-dependent order–disorder transition of Mpda cations and the lattice expansion of inorganic skeleton [43]. At room temperature, it crystallizes in a polar space group *Pn* (no. 7) and adopts a typical 2D DJ motif with alternating Mpda organic layers and [PbBr_4_]^∞^ inorganic slabs, as shown in Fig. 1a. This non-centrosymmetric structure is further corroborated by a pronounced SHG signal, with intensity comparable to the reference KH_2_PO_4_ (KDP), as plotted in Fig. 1b. The organic diamine cations are vertically anchored to two adjacent inorganic slabs through strong N-H···Br hydrogen bonds, which constitute 77.3% of all intermolecular forces as visualized by the Hirshfeld surface analysis (Fig. 1c, d) [47]. These strong interactions not only enable Mpda to be asymmetrically aligned in the interlayer space but also induce large distortion within the inorganic octahedral layer (Fig. S1), synergistically displacing the positive and negative charge centers and thus generating out-of-plane polarization along the *c*-axis in (Mpda)PbBr_4_. The spontaneous polarization strength is calculated to be 51.99 *μ*C cm^−2^ based on the point charge model, as listed in Table S1. This strong out-of-plane polarization can establish a robust built-in electric field along this direction, suggesting its great potential for constructing vertical optoelectronic devices.

Light-yellow microcrystals of (Mpda)PbBr_4_ were grown from the hot HBr solution containing stoichiometric lead acetate trihydrate and Mpda *via* a slow cooling process. Their phase purity was confirmed by powder XRD patterns (Fig. S2). The TG curve of (Mpda)PbBr_4_ powders shows a high decomposition temperature of 598 K (Fig. S3), revealing its excellent thermal stability and great suitability for hot-pressing process. Figure 1e shows the UV–*Vis* absorption spectrum of (Mpda)PbBr_4_, in which a sharp cutoff at 434 nm is observed. According to the Tauc equation, its optical bandgap (*E*_g_) can be determined to be 2.88 eV [48]. To have a deeper understanding of its electronic structure, DFT calculations were performed. As shown in Fig. 1f, (Mpda)PbBr_4_ exhibits a direct bandgap featuring the conduction band minimum (CBM) and valence band maximum (VBM) located at the same point of *G*. The calculated bandgap is 2.35 eV, slightly smaller than the experimental result, which can be ascribed to the inherent drawback of Perdew–Burke–Ernzerhof (PBE) exchange and correlation functional [49]. The partial density of states (DOS) profiles suggest that the VBM is primarily governed by Br 4*p* orbitals, while the CBM is mainly stemming from Pb 6*p* orbitals, indicating that the bandgap and electronic structure are chiefly determined by the lead bromide inorganic framework. The UPS spectrum was measured to determine the energy levels of (Mpda)PbBr_4_, as presented in Fig. 1g. From the secondary electron cutoff edge of this spectrum, its work function is 3.82 eV, and the valence band is located at 2.01 eV below the Fermi level (*E*_F_). Combined with the optical bandgap, the energy-level diagram displays that the CBM, VBM, and *E*_F_ are located at −2.95, −5.83, and −3.82 eV (Fig. 1h). The *E*_F_ value is 0.57 eV higher than middle position of its forbidden band (*E*_i_ = −4.39 eV), indicative of n-type electronic property of (Mpda)PbBr_4_. This energy-level structure can provide guidance for preparing (Mpda)PbBr_4_ semiconductor device and optimizing its device structure.

### High-Quality Wafer Fabrication and Ferroelectricity

**Fig. 2:**
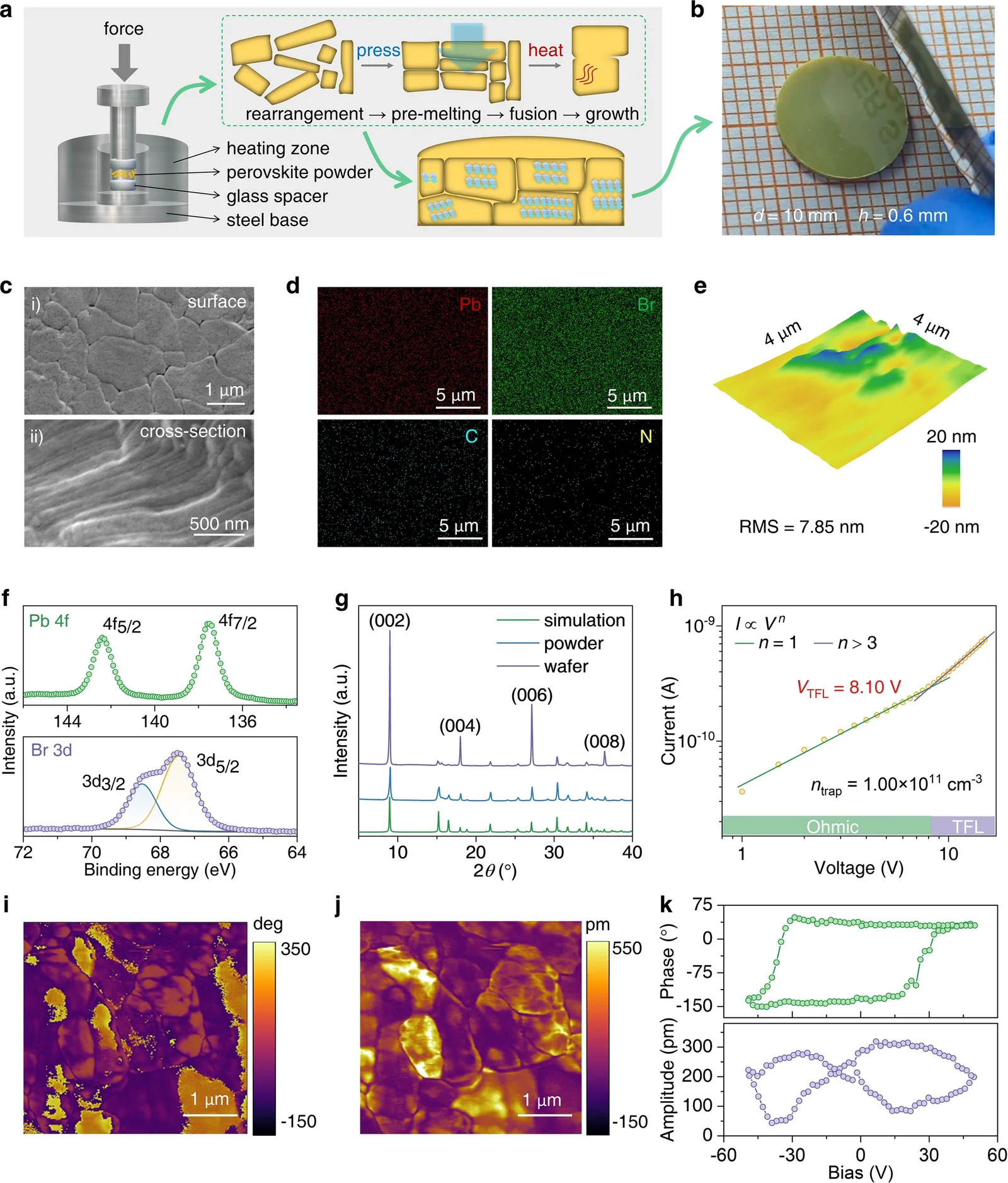
(Mpda)PbBr_4_ wafer fabrication and ferroelectricity. **a** Schematic diagram for (Mpda)PbBr_4_ polycrystalline wafer fabrication using a hot-pressing process. **b** Photograph of (Mpda)PbBr_4_ wafer, showing a mirror-like surface. **c, d** Surface and cross-section SEM images of (Mpda)PbBr_4_ wafer and corresponding EDS mappings for Pb, Br, C, and N elements. **e** AFM image of (Mpda)PbBr_4_ wafer surface, displaying an RMS value of 7.85 nm. **f** High-resolution XPS spectra of Pb 4f and Br 3d in (Mpda)PbBr_4_. **g** XRD patterns of (Mpda)PbBr_4_ powders and wafer, respectively, using the simulated pattern as reference. **h** Logarithmic *I*-*V* curve of (Mpda)PbBr_4_ wafer using the SCLC method, giving a trap density of *n*_trap_ = 1.00 × 10^11^ cm^−3^. **i–j** Vertical PFM phase and amplitude images for the (Mpda)PbBr_4_ film. **k** Vertical PFM phase (up) and amplitude (down) as a function of DC bias, showing local hysteresis loops

To circumvent the challenges and long period associated with growing large-sized single crystals of 2D MHPs, here we turn to fabricate high-quality polycrystalline wafers *via* a scalable hot-pressing method [44, 50]. Figure 2a schematically illustrates the process of wafer tableting. Well-ground (Mpda)PbBr_4_ microcrystal powders were used as the precursor to achieve high phase purity. Under simultaneous heating and uniaxial pressure, the powders undergo a dynamic process of rearrangement, pre-melting, fusion, and grain growth, finally forming densified, pinhole-free wafers. In this process, three parameters, *e.g.*, temperature, pressure, and time, were systematically optimized (Fig. S4) to prevent decomposition and ensure high quality and preferred orientation texture, yielding an optimal combination of temperature at 170 °C, pressure at 300 MPa, and holding time for 60 min. As shown in Fig. 2b, a dark yellow wafer with a diameter of 10 mm and thickness of 0.6 mm was successfully fabricated, which exhibits a highly smooth and mirror-like surface, indicating that it is significantly densified with high quality. The micromorphology of (Mpda)PbBr_4_ wafer was studied using SEM, as shown in Fig. 2c. The surface image reveals a dense morphology constructed by well-defined large grains (1~2 *μ*m) without visible voids, while the cross-sectional SEM image displays a distinct layered texture, suggesting a preferred orientation during hot-pressing [44]. Corresponding EDS mappings show that all elements such as Pb, Br, C, and N are uniformly distributed within this wafer (Fig. 2d). Its surface roughness was characterized by AFM, with a root-mean-square average (RMS) of only 7.85 nm, further verifying the high quality of (Mpda)PbBr_4_ wafer (Fig. 2e). XPS survey of (Mpda)PbBr_4_ wafer clearly shows the containing of Pb, Br, C, and N peaks (Fig. S5). High-resolution XPS spectra for Pb 4*f* and Br 3*d* are plotted in Fig. 2f, in which these detected peaks can be attributed to characteristic signals of Pb^2+^ and Br^−^ species, respectively. XRD analysis of (Mpda)PbBr_4_ wafer shows the characteristic peaks consistent with the standard pattern (CCDC 2277171) [43], confirming its phase purity. Comparing to the XRD pattern of (Mpda)PbBr_4_ powders, it is intriguing that diffraction peaks of (00*l*) planes are significantly intensified in wafer, revealing that an obvious horizontal orientation occurred in the rearrangement and growth of micrograins during hot-pressing tableting. As is well known, the 2D MHP cell occupies a minimum volume when all planes are parallel to the horizontal level. Consequently, this phenomenon can be ascribed to more (00*l*) planes becoming parallel to the substrate, thereby minimizing the occupied volume upon external pressure [44–46]. Such a strongly preferred (00*l*) orientation indicates that the intrinsic, out-of-plane polar *c*-axes of the vast majority of (Mpda)PbBr_4_ micrograins are aligned along the vertical direction, thereby providing a critical structural basis for acquiring a net macroscopic polarization in the polycrystalline wafer. The trap-state density of (Mpda)PbBr_4_ polycrystalline wafer was assessed using the space-charge-limited-current (SCLC) test on an Ag/(Mpda)PbBr_4_/Ag vertical device. As plotted in Fig. 2h, the logarithmic *I*-*V* curve exhibits distinct Ohmic (*n* = 1) and trap-filling limit (TFL, *n* > 3) regions, yielding a TFL threshold voltage (*V*_TFL_) of 8.10 V. According to the following formula [5, 13]:

1 $${n}_{\mathrm{t}\mathrm{r}\mathrm{a}\mathrm{p}}=\frac{2{\varepsilon}_{\mathrm{r}}{\varepsilon}_{0}{V}_{\mathrm{T}\mathrm{F}\mathrm{L}}}{q{L}^{2}}$$

in which *n*_trap_ denotes the trap density of this wafer, *L* is the thickness, *q* is the unit charge, and *ε*_r_ and *ε*_0_ represent the relative dielectric constant of sample and the vacuum dielectric constant, respectively. The average *ε*_r_ value for (Mpda)PbBr_4_ wafer is measured to be 40 at room temperature, as shown in Fig. S6. Thus, *n*_trap_ is calculated to be 1.00×10^11^ cm^−3^, which is slightly higher than values reported in high-quality 3D MHP wafers, like MAPbBr_3_ (MA = methylamine, 6.7 × 10^10^ cm^−3^) [51], and FAPbBr_3_ (2.31×10^10^ cm^−3^) [52], but it is significantly lower than those of 2D MHP films, such as (BA)_2_PbBr_4_ (BA = butylamine, 8.49×10^13^ cm^−3^) [53] and (*p*−FC_6_H_4_C_2_H_4_NH_3_)_2_PbI_4_ (4.37×10^15^ cm^−3^) [54]. This result indicates that the hot-pressed 2D (Mpda)PbBr_4_ wafer has relatively high quality, contributing to achieving high optoelectronic performance.

To investigate the ferroelectricity of (Mpda)PbBr_4_ in a polycrystalline form, the PFM measurement was performed on the spin-coated film at room temperature [55, 56]. The surface topography of (Mpda)PbBr_4_ film is shown in Fig. S7, and corresponding vertical (out-of-plane) PFM phase and amplitude images are demonstrated in Fig. 2i, j. Clearly, the observed phase signals with yellow and purple colors have a contrast of about 180°, which correspond to different domains with upward and downward polarization directions in the film [57]. Simultaneously, the PFM amplitude image indicates that these domains have different piezoresponse. Further, the local PFM spectroscopic measurements were carried out to confirm that the polarization is switchable. As shown in Fig. 2k, the typical phase–voltage hysteresis loop and the butterfly-shaped amplitude–voltage curve suggest that the ferroelectric polarization is electrically switchable, confirming the ferroelectric nature of (Mpda)PbBr_4_ in a polycrystalline state.

**Fig. 3:**
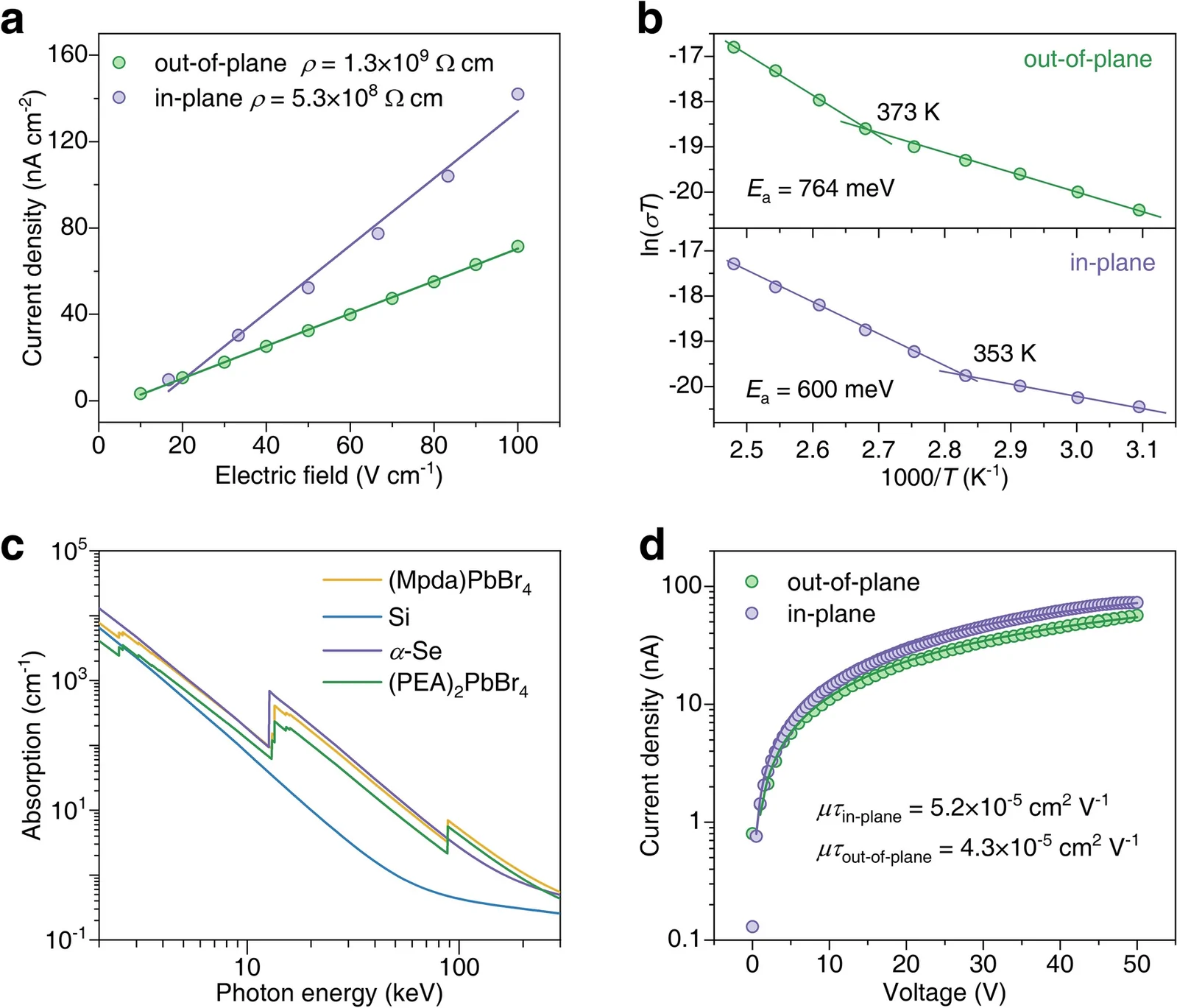
Electrical properties and X-ray absorption of (Mpda)PbBr_4_ wafer. **a** Bulk resistivities of (Mpda)PbBr_4_ wafer in the in-plane (lateral) and out-of-plane (vertical) directions, respectively. **b** Temperature-dependent conductivities of (Mpda)PbBr_4_ wafer along the in-plane and out-of-plane directions. **c** X-ray absorption spectra of (Mpda)PbBr_4_, Si, *α*-Se, and (PEA)_2_PbBr_4_. **d** Voltage-dependent photocurrent of (Mpda)PbBr_4_ wafer under X-ray irradiation along the in-plane and out-of-plane directions

Subsequently, the electrical properties of (Mpda)PbBr_4_ wafer along the out-of-plane and in-plane directions were systematically evaluated. It should be noted that the measured in-plane and out-of-plane electric properties here refer to the lateral and vertical directions of the (Mpda)PbBr_4_ wafer, respectively. Owing to the preferred grain orientation, these properties are dominated by the intrinsic transport along the crystallographic *ab*-plane and *c*-axis. Therefore, to connect the macroscopic properties of (Mpda)PbBr_4_ wafer with its intrinsic crystal structure, we also used the terms “in-plane” and “out-of-plane” for the wafer device. Figure S8 schematically illustrates the corresponding device configurations. Figure 3a presents the bulk resistivity (*ρ*) of (Mpda)PbBr_4_ wafer along the in-plane and out-of-plane directions, respectively. Obviously, a higher *ρ* value (1.3×10^9^ Ω cm) is obtained in the out-of-plane direction, further supporting the preferred horizontal orientation of micrograins. Temperature-dependent conductivity curves of (Mpda)PbBr_4_ wafer along in-plane and out-of-plane directions were, respectively, measured under a bias of 20 V (Fig. 3b), which can be used to estimate the activation energy (*E*_a_) for ion migration in this wafer. According to the Nernst–Einstein equation [25, 58]:

2 $$\sigma =\left(\frac{{\sigma}_{0}}{T}\right)\mathrm{exp}\left(\frac{-{E}_{\mathrm{a}}}{{k}_{\mathrm{B}}T}\right)$$

where *σ* is the conductivity at a given temperature *T*, *σ*_0_ is a constant, and *k*_B_ is the Boltzmann constant. By fitting the relationship between ln(*σT*) and 1000/*T*, it is clear that there exists a transition from electronic to ionic conductivity in both directions as the temperature increases. The fitting results show that *E*_a_ for the out-of-plane direction (764 meV) is higher than that of the in-plane direction (600 meV), indicating a more effective suppression on ion migration. For X-ray detection, efficient attenuation and charge collection are crucial. Figure 3c shows the X-ray absorption spectra over a wide energy range of 2~233 keV of (Mpda)PbBr_4_ and some typical X-ray detection semiconductors, *e.g.*, Si, *α*-Se, and (PEA)_2_PbBr_4_ (PEA = phenylethylamine), which are simulated based on the XCOM database of the National Institute of Standards and Technology (NIST) [59]. (Mpda)PbBr_4_ possesses a high linear attenuation coefficient, exceeding those of Si and (PEA)_2_PbBr_4_ and approaching that of *α*-Se, revealing its good X-ray attenuation ability. Figure S9 presents the attenuation efficiencies versus thickness of the above materials for 20 keV X-ray photons (the energy that we used for X-ray detection performance evaluation). As seen, at a thickness of 0.6 mm, (Mpda)PbBr_4_ can completely absorb the incident X-rays (99.99%), which is significantly higher than that of Si (44.81%). The charge collection efficiency, quantified by the mobility-lifetime product (*μτ*), was extracted by fitting *I*-*V* curves of (Mpda)PbBr_4_ wafer under X-ray irradiation (here the dose rate is 60.1 *μ*Gy s^−1^) with the Hecht equation [32, 60]:

3 $$I=\frac{{I}_{0}\mu \tau V}{{L}^{2}}\left[1-\mathrm{exp}\left(-\frac{{L}^{2}}{\mu \tau V}\right)\right]$$

in which *I* is the photocurrent, *I*_0_ represents the saturated photocurrent, *L* denotes the electrode spacing, and *V* is the voltage. As shown in Fig. 3d, *μτ* values in the in-plane and out-of-plane directions are estimated to be 5.2×10^−5^ and 4.3×10^−5^ cm^2^ V^−1^, respectively. Such a difference can be ascribed to the smoother carrier transport provided by the continuous [PbBr_6_] octahedra in the inorganic layer [44, 61]. Even so, the out-of-plane *μτ* value remains comparable to those of some reported 2D MHPs, such as (BZA)_2_(MA)Pb_2_Br_7_ (2.2×10^−5^ cm^2^ V^−1^, BZA = benzylamine) [62], (*R*−MPA)_2_PbBr_4_ (5.53×10^−5^ cm^2^ V^−1^, *R*-MPA = *R*-methylphenethylamine) [63], and (F-PEA)_3_BiI_6_ (8.3×10^−5^ cm^2^ V^−1^, F-PEA = 4-fluorophenethylamine) [44], and is orders of magnitude higher than that of commercial *α*-Se film (~ 10^−7^ cm^2^ V^−1^), suggesting its strong potential for direct X-ray detection.

### Macroscopic Polarization and Built-In Electric Field

**Fig. 4:**
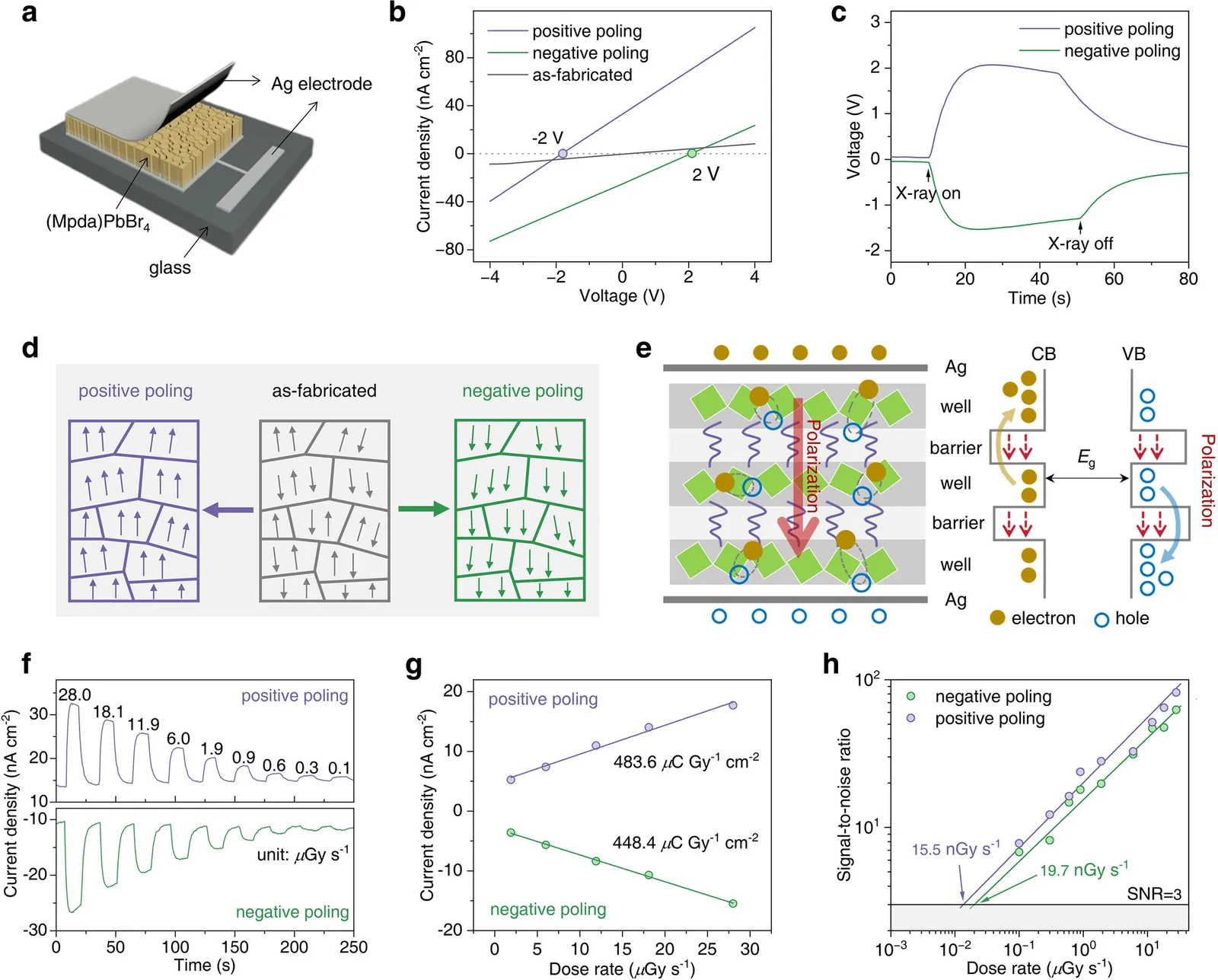
Macroscopic polarization-induced built-in electric field and self-powered X-ray detection in (Mpda)PbBr_4_ wafer. **a** Schematic illustration of the vertical Ag/(Mpda)PbBr_4_ wafer/Ag device. **b** *J*-*V* curves of (Mpda)PbBr_4_ wafer as fabricated and after negatively or positively poled under X-ray irradiation. **c** *V*-*t* curves of (Mpda)PbBr_4_ device after negative or positive poling, exhibiting stable open-circuit voltage upon X-ray on/off. **d** A schematic displaying the polarization state of (Mpda)PbBr_4_ wafer as fabricated and after negative or positive poling. **e** Enhanced interlayer carrier transport in (Mpda)PbBr_4_ under the influence of out-of-plane polarization. **f** *J*-*t* curves of positively or negatively poled (Mpda)PbBr_4_ device under various X-ray dose rates at zero bias. **g, h** Sensitivities and detection limits of positively or negatively poled (Mpda)PbBr_4_ detector under self-powered mode

To further study the influence of macroscopic out-of-plane polarization on the photoresponse, a vertical device of Ag/(Mpda)PbBr_4_ wafer/Ag was fabricated by depositing Ag paste on its top and bottom sides (Figs. 4a and S10). Prior to measurement, the wafer was subjected to a poling process (±200 V for 5 min) to align the polarized domains. After positive poling, a large open-circuit voltage (*V*_oc_) value of −2 V and a short-circuit current density (*J*_sc_) value of 33.01 nA cm^−2^ were observed in the current density–voltage (*J*-*V*) curve of (Mpda)PbBr_4_ device under X-ray irradiation (60.10 *μ*Gy s^−1^), as shown in Fig. 4b. Reversing the poling electric field (negative poling) switches the sign of both *V*_oc_ and *J*_sc_ accordingly (2 V and −25.17 nA cm^−2^), demonstrating a typical and electrically switchable BPVE characteristic [43, 45, 57]. The stability of this bulk photovoltage is shown in the *V*-*t* curves under alternating poling states (Fig. 4c). This phenomenon can be ascribed to the alignment of initially randomized polarization directions within individual micrograins with the assistance of external electric field, as shown in Fig. 4d. As the wafer is fabricated, even though the *c*-axes (intrinsic polar axis) of most micrograins are aligned along the vertical direction, their spontaneous polarization directions can be upward or downward, thus resulting in negligible net macroscopic polarization. After positive or negative poling, the polarization directions of most textured, individual grains are oriented consistently along the external electric field, hence establishing a significant net out-of-plane macroscopic polarization across the wafer. This aligned polarization further creates a strong built-in electric field that can efficiently separate X-ray-generated carriers and drive their out-of-plane transport (Fig. 4e) [26, 36], thereby exhibiting a stable bulk photovoltage. Benefiting from this intriguing built-in electric field, the Ag/(Mpda)PbBr_4_ wafer/Ag detector can operate in self-powered mode without external bias. As shown in Fig. 4f, both poling states yield clear X-ray photocurrent responses at zero bias. The magnitude and direction of the photocurrent density depend on the X-ray dose rate and applied direction of the poling electric field. With the X-ray dose rate gradually increasing from 0.1 to 28.0 *μ*Gy s^−1^, the corresponding photocurrent density increases from 15.80 to 32.50 nA cm^−2^, showing a good linear response characteristic. To quantitatively evaluate the X-ray detection performance of poled (Mpda)PbBr_4_ wafer, two key parameters in terms of sensitivity and detection limit were introduced. The sensitivity can be calculated by Eq. (4) [13]:

4 $$S=\frac{{J}_{\mathrm{p}\mathrm{h}} - {J}_{\mathrm{d}}}{D}$$

where *J*_ph_ and *J*_d_ are current densities under X-ray irradiation and in the dark, respectively, and *D* is the X-ray dose rate. According to the International Union of Pure and Applied Chemistry (IUPAC), the detection limit is defined as the X-ray dose rate that produces a signal with signal-to-noise ratio (SNR) equal to 3. The SNR value is determined by Eq. (5) [30, 32]:

5 $$\mathrm{S}\mathrm{N}\mathrm{R}=\frac{\overline{{J }_{\mathrm{p}\mathrm{h}}}-\overline{{J }_{\mathrm{d}}}}{\sqrt{\frac{1}{n}{\sum}_{1}^{n}{\left({J}_{i}-\overline{{J }_{\mathrm{p}\mathrm{h}}}\right)}^{2}}}$$

in which $\overline{{J }_{\mathrm{p}\mathrm{h}}}$ is the average photocurrent density under X-ray irradiation, $\overline{{J }_{\mathrm{d}}}$ is the average dark current density, and *J*_*i*_ is the photocurrent density of *i*^th^ data dot. As shown in Fig. 4g, the device achieves sensitivities of 483.6 (positive poling) and 448.4 *μ*C Gy^−1^ cm^−2^ (negative poling), respectively, under self-powered mode. These values surpass those of many reported self-powered 2D MHP detectors, *e.g.*, (BZA)_2_(MA)Pb_2_Br_7_ (97.9 *μ*C Gy^−1^ cm^−2^) [62], EA_4_Pb_3_Br_10_ (203.3 *μ*C Gy^−1^ cm^−2^, EA = ethylamine) [45], and (TFMPEA)_4_AgBiI_8_ (184 *μ*C Gy^−1^ cm^−2^, TFMPEA = 2-(4-trifluoromethyl-phenyl)-ethylamine) [38], underscoring the efficacy of the polarization-induced built-in electric field in facilitating interlayer carrier transport. Figure 4h displays signal-to-noise ratios (SNRs) of positively or negatively poled (Mpda)PbBr_4_ wafer under varying X-ray dose rates, from which their detection limits are determined to be 15.5 and 19.7 nGy s^−1^, respectively, revealing the capability of (Mpda)PbBr_4_ wafer to detect weak X-rays.

### X-Ray Detection and Imaging

**Fig. 5:**
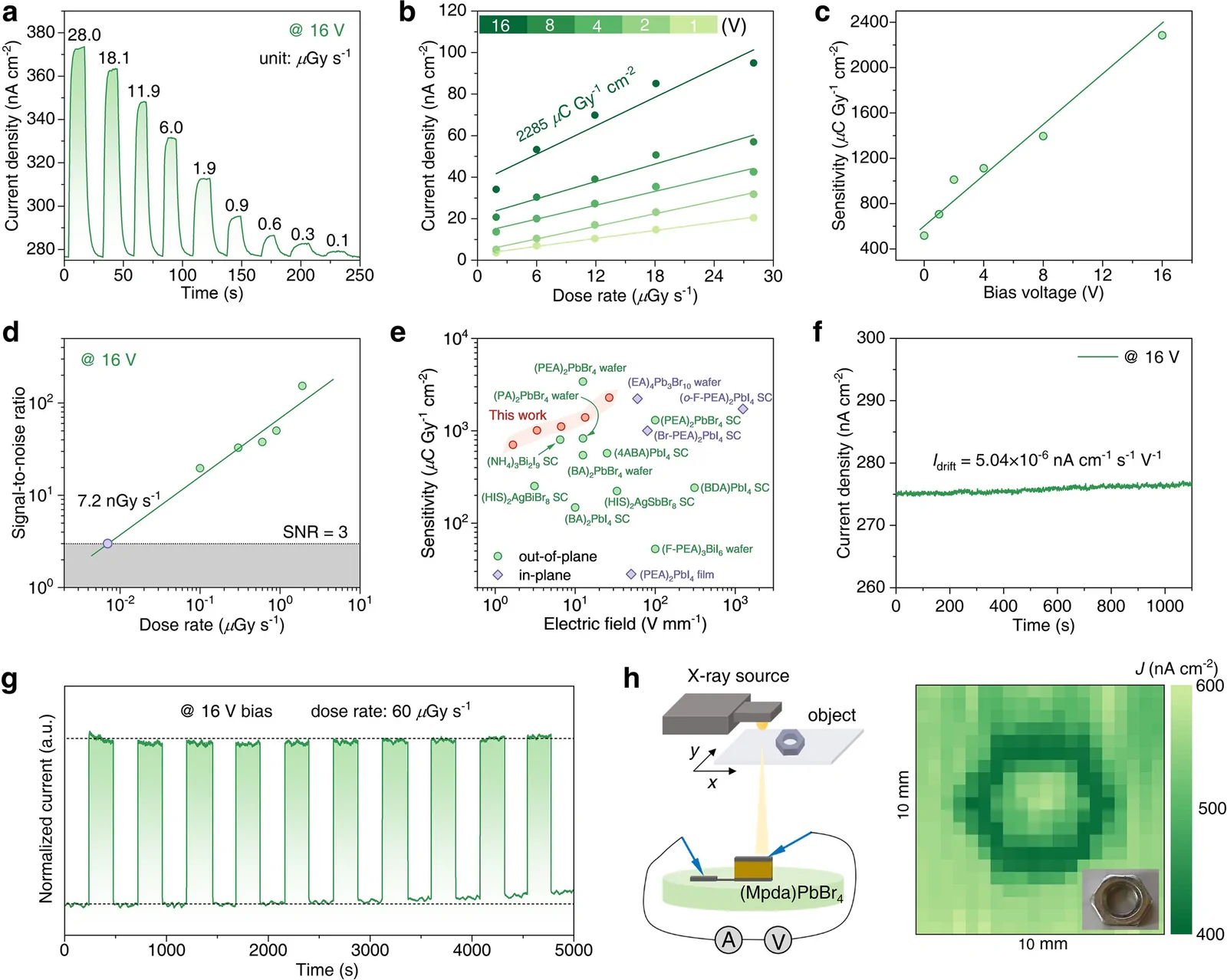
X-ray detection and imaging performance. **a** *J*-*t* curves of positively poled (Mpda)PbBr_4_ wafer detector under various X-ray dose rates (0.1 – 28.0 *μ*Gy s^−1^) at 16 V bias. **b** X-ray-induced current density as a function of dose rate under various bias voltages (*e.g.*, 1, 2, 4, 8, and 16 V). Linear fitting their slopes obtains the voltage-dependent sensitivity of (Mpda)PbBr_4_ device. **c** Sensitivities of (Mpda)PbBr_4_ detector at various bias voltages. **d** SNRs of (Mpda)PbBr_4_ detector under various X-ray dose rates at 16 V bias, deriving a LoD of 7.2 nGy s^−1^. **e** Comparison of the X-ray detection sensitivity of (Mpda)PbBr_4_ wafer and some reported 2D MHPs. **f** Dark current drift of (Mpda)PbBr_4_ wafer device at 16 V bias. **g** Normalized current–time curves of detector under continuous X-ray on/off, showing excellent long-term operational stability. **h** A schematic illustration of the X-ray imaging system based on wafer (left), and the photograph and corresponding X-ray image of a metal nut

We further evaluated the systematic X-ray detection performance of the macroscopically polarized 2D MHP wafer. Upon applying a small bias (1 V), the *J*-*t* curve of the poled (Mpda)PbBr_4_ wafer shows a significantly enhanced photocurrent response compared to the as-fabricated one (Fig. S11), confirming that the built-in electric field, arising from aligned polarization, synergistically reinforces carrier extraction under an external electric field. Further, *J*-*t* curves of the polarized wafer under different external bias voltages (*e.g.*, 2, 4, 8, and 16 V) were measured (Figs. S12, S13, and 5a). As shown in Fig. 5a, this device exhibits a linear and sensitive photocurrent increase across a broad range of X-ray dose rates, with a clear response even at a low dose rate of 100 nGy s^−1^, confirming its excellent X-ray detection performance under external bias operation. Figure 5b shows the change of X-ray-induced current densities with increasing X-ray dose rates, from which the sensitivities under different external bias can be derived. The sensitivity gradually grows with the increasing bias voltages due to the more efficient charge collection under larger electric field [13, 40], reaching 2285 *μ*C Gy^−1^ cm^−2^ at 16 V bias (Fig. 5c). Figure 5d shows SNRs under various X-ray dose rates at 16 V bias. Notably, a high SNR of 19.66 is obtained even at a low dose rate of 100 nGy s^−1^. Fitting the relationship between SNR and dose rate gives an ultralow detection limit of 7.2 nGy s^−1^, which is about 763-fold lower than that required for regular medical imaging (5500 nGy s^−1^). Furthermore, such a low detection limit can be achieved at different applied biases (Fig. S14), indicating its suitability for low-dose applications. A comprehensive comparison with many recently reported 2D MHP X-ray detectors (Table S2 and Fig. 5e) highlights that the (Mpda)PbBr_4_ wafer achieves not only high sensitivity but also an ultralow detection limit, revealing its strong potential for use in high-performance X-ray detection and imaging.

The operational stability is another key parameter of X-ray detectors. To quantify this, the dark current drift (*I*_drift_) of (Mpda)PbBr_4_ wafer detector is calculated by Eq. (6) [7, 32]:

6 $${I}_{\mathrm{d}\mathrm{r}\mathrm{i}\mathrm{f}\mathrm{t}}=\frac{{J}_{t}-{J}_{0}}{E\times t}$$

where *J*_t_ and *J*_0_ represent dark current density at time *t* and 0, *E* denotes the electric field intensity, and *t* is the operating time. As shown in Fig. 5f, the dark current density of (Mpda)PbBr_4_ wafer maintains highly stable over time, yielding a small *I*_drift_ of 5.04×10^−6^ nA cm^−1^ V^−1^ s^−1^. This value is comparable to those of reported high-quality MHP wafers, such as MAPbBr_3_ (2.36×10^−5^ nA cm^−1^ V^−1^ s^−1^) [64], MAPbI_3_ (1.31×10^−4^ nA cm^−1^ V^−1^ s^−1^) [65], and EA_4_Pb_3_Br_10_ (1.04×10^−6^ nA cm^−1^ V^−1^ s^−1^) [45], revealing the excellent working stability of (Mpda)PbBr_4_ wafer. Furthermore, we also tested the operational stability of the device by exposing it to continuous X-ray on/off cycles with a high dose rate of 60.1 *μ*Gy s^−1^ (Fig. 5g). As seen, a stable and repeatable X-ray response can be acquired even after long-term operation, indicating its great potential for practical application. Finally, we evaluated the imaging capability of (Mpda)PbBr_4_ wafer detector in a single-pixel imaging mode, as illustrated in Fig. 5h (left). In this work, a metal nut was selected as the object for imaging, and the X-ray dose rate and operational voltage were set to 60.1 *μ*Gy s^−1^ and 20 V, respectively. As shown in Fig. 5h, a well-defined, high-contrast image of this nut was successfully reconstructed by mapping position-dependent response current densities with color scales. This result suggests that the 2D (Mpda)PbBr_4_ polycrystalline wafer is suitable for X-ray imaging and exhibits the potential for use in areas like medical imaging or nondestructive testing.

## Conclusions

In summary, we successfully achieved macroscopic out-of-plane polarization in a centimeter-sized 2D ferroelectric perovskite polycrystalline wafer, (Mpda)PbBr_4_, via a facile hot-pressing strategy, which circumvents the difficulty of large-size crystal growth and enhances the interlayer carrier transport, thus achieving efficient X-ray detection. During processing, (Mpda)PbBr_4_ micrograins adopt a horizontal orientation, enabling the vertical alignment of their polarization axes. Subsequent electrical poling yields a macroscopic polarization within the wafer, thus generating a robust, switchable built-in electric field. This field drives the efficient separation and interlayer transport of photogenerated carriers, manifesting as a pronounced bulk photovoltaic effect (~ 2 V). As a result, the vertical X-ray detector based on poled wafer operates effectively in a self-powered mode, achieving a sensitivity exceeding 448 *μ*C Gy^−1^ cm^−2^. The synergy between the built-in and an external electric field (16 V) further enhances the detection performance, delivering a high sensitivity of 2285 *μ*C Gy^−1^ cm^−2^ and an ultralow detection limit of 7.2 nGy s^−1^, which surpasses the performance of many reported 2D perovskite-based detectors. The device also demonstrates excellent operational stability, with a minimal baseline drift of 5.04×10^−6^ nA cm^−1^ V^−1^ s^−1^, and showcases high-resolution imaging capability. This study establishes that engineering macroscopic out-of-plane polarization in polycrystalline wafer is a promising pathway to overcome inefficient interlayer carrier transport, providing insights into future design and fabrication of high-performance, flat-panel X-ray detectors based on 2D hybrid perovskites.

## Supplementary Information

Below is the link to the electronic supplementary material.

Supplementary file1 (DOCX 11251 kb)
