# Supplementary material for: Aligning Out-of-Plane Polarization in 2D Ferroelectric Perovskite Wafer Boosts Interlayer Carrier Transport for Efficient X-Ray Detection
Source: Nanomicro Lett. 2026 Oct 15;19:54. doi: 10.1007/s40820-026-02352-z (PMC13669269; doi:10.1007/s40820-026-02352-z)
Supplement: Supplementary file 1 [file 40820_2026_2352_MOESM1_ESM.docx]

Supporting Information for

**Aligning Out-of-Plane Polarization in 2D Ferroelectric Perovskite Wafer Boosts Interlayer Carrier Transport for Efficient X-Ray Detection**

Chenhao Deng^1^, Shihai You^1,^ *, Haiqing Zhong^2^, Xiankan Zeng^1^, Xiaolong Hao^1^, Jiasheng Xie^1^, Zheng Zhou^1^, Zhangjian Jin^1^, Huang Ye^2^, Lijun Xu^2^, Jianbo Wu^3^, Panpan Dong^1^, Wen Li^1^, Xingxing Jiao^1^, Weiqing Yang^1,^ *

^1^ Key Laboratory of Advanced Technologies of Materials (Ministry of Education), School of Materials Science and Engineering, Research Institute of Frontier Science, Southwest Jiaotong University, Chengdu 610031, P. R. China

^2^ State Key Laboratory of Functional Crystals and Devices, Fujian Institute of Research on the Structure of Matter, Chinese Academy of Sciences, Fuzhou 350002, P. R. China

^3^ Department of Materials Science and Engineering, City University of Hong Kong, Hong Kong SAR 999077, P. R. China

* Corresponding authors. E-mail: [shihai.you@swjtu.edu.cn](mailto:shihai.you@swjtu.edu.cn) (Shihai You); [wqyang@swjtu.edu.cn](mailto:wqyang@swjtu.edu.cn) (Weiqing Yang)

**S1 Supporting Figures**

**Fig. S1** a) Inorganic layer of (Mpda)PbBr_4_ constructed by distorted [PbBr_6_] octahedra through corner-sharing. b) Pb-Br bond lengths in two distinct [PbBr_6_] octahedra (labeled with 1 and 2).

According to the formula [S1],

$$\begin{aligned} \text{∆d=}\frac{\text{1}}{\text{6}}\sum_{\text{1}}^{\text{6}} \left| \frac{\text{d}_{\text{i}}\text{-}\text{d}_{\text{0}}}{\text{d}_{\text{0}}} \right|^{\text{2}}\#(S1) \end{aligned}$$

in which Δ*d* denotes the degree of distortion, and *d_i_* and *d*_0_ are individual and average Pb-Br bond lengths. The distortion degrees of the two [PbBr_6_] octahedra are calculated to be 30×10^-5^ (1) and 25×10^-5^ (2), respectively, showing they are highly distorted.

**Fig. S2** Comparison of simulated and experimental powder XRD patterns of (Mpda)PbBr_4_.

**Fig. S3** TG curve of (Mpda)PbBr_4_ powders under N_2_ atmosphere.

**Fig. S4** XRD patterns of (Mpda)PbBr_4_ wafers under different hot-pressing parameters: a) holding time, b) temperature, c) pressure, and d) different pressure with temperature of 170 °C. This systematically study gives the optimal fabrication parameters: 170 °C, 300 MPa, and 60 min.

**Fig. S5** XPS spectrum of (Mpda)PbBr_4_ scanning from 0 to 1200 eV.

**Fig. S6** Frequency-dependent relative dielectric constant of (Mpda)PbBr_4_ wafer.

**Fig. S7** The PFM topography of (Mpda)PbBr_4_ film.

**Fig. S8** Schematic configuration of the (a) out-of-plane (vertical) and (b) in-plane (lateral) directions in (Mpda)PbBr_4_ wafer.

**Fig. S9** Attenuation efficiency of (Mpda)PbBr_4_, Si, *α*-Se, and (PEA)_2_PbBr_4_ for 20 keV X-rays versus thickness.

**Fig. S10** Photograph of Ag/(Mpda)PbBr_4_ wafer/Ag vertical device.

**Fig. S11** Comparison of photocurrent response of (Mpda)PbBr_4_ wafer as fabricated and after poling under X-ray irradiation (external bias of 1 V), showing a significant enhancement.

**Fig. S12** *J*-*t* curves of (Mpda)PbBr_4_ wafer detector under X-ray irradiation operated under different external bias voltages (*e.g.*, 1, 2, 4, and 8 V).

**Fig. S13** *J*-*t* curves of (Mpda)PbBr_4_ wafer detector under low dose rate X-ray irradiation.

**Fig. S14** Detection limits of (Mpda)PbBr_4_ wafer detector operated at different bias voltages.

**S2 Supporting Tables**

**Table S1** The atomic coordinates of Pb and N in crystal structure of (Mpda)PbBr_4_ collected at 293 K [S2]

| Atom | Atomic coordinate | | Coordinate of charge center |
| --- | --- | --- | --- |
| Pb | Pb1^i^ (0.74103, 0.60812, 0.04472) | Pb1^ii^ (0.24103, 0.39188, 0.54472) | (0.49103, 0.5, 0.29472) |
|  | Pb2^i^ (0.23971, 0.09787, 0.04251) | Pb2^ii^ (0.73971, 0.90213, 0.54251) | (0.48971, 0.5, 0.29251) |
| N | N1^i^ (0.75060, 0.17830, 0.93020) | N1^ii^ (0.25060, 0.82170, 0.43020) | (0.50060, 0.5, 0.68020) |
|  | N2^i^ (0.42340, 0.99500, 0.71321) | N2^ii^ (0.92340, 0.00500, 0.21321) | (0.67340, 0.5, 0.46321) |
|  | N3^i^ (0.24680, 0.52590, 0.92020) | N3^ii^ (0.74680, 0.47410, 0.42020) | (0.49680, 0.5, 0.67020) |
|  | N4^i^ (0.93550, 0.50800, 0.71320) | N4^ii^ (0.43550, 0.49200, 0.21320) | (0.68550, 0.5, 0.46320) |

According to the point charge model, we assume that the centers of the positive charges of Mpda cation and the centers of negative charges of [PbBr_6_] octahedra are locating at N and Pb atoms, respectively.

Thus, spontaneous polarization intensity (*P*_s_) along the *c*-axis,

*P*_s_ = (*q*_N_*r*_N_ + *q*_Pb_*r*_Pb_) × *Z* × *c* / *V*

= [(e × 0.68020) × 2 + (e × 0.46321) × 2 + (e × 0.67020) × 2 + (e × 0.46320) × 2 + (-2e × 0.29472) ×2 + (-2e × 0.29251) ×2] × 20.13716 ×10^-10^ / (1366.35 ×10^-30^)

= 1.6 × 10^-19^ × 2.2047 × 20.13716 / 1366.35 × 10^20^ C m^-2^

**Table S2** Detection performance comparison between (Mpda)PbBr_4_ wafer and some recently reported 2D MHP X-ray detectors

| Compound | Electric field (V mm^-1^) | Sensitivity  (*µ*C Gy^-1^ cm^-2^) | Detection limit  (nGy s^-1^) | Form | Direction | Refs |
| --- | --- | --- | --- | --- | --- | --- |
| (Mpda)PbBr_4_ | 0 | 483.6 | 15.5 | wafer | out-of-plane | This work |
|  | 1.67 | 705.4 | 18.6 | wafer |  |  |
|  | 3.33 | 1011.5 | 12.6 | wafer |  |  |
|  | 6.67 | 1112.1 | 10 | wafer |  |  |
|  | 13.33 | 1395.5 | 9.6 | wafer |  |  |
|  | 26.67 | 2285 | 7.2 | wafer |  |  |
| (F-PEA)_3_BiI_6_ | 100 | 52.6 | 30 | wafer | out-of-plane | [S3] |
| (PEA)_2_PbI_4_ | 50 | 28.1 | - | film | in-plane | [S4] |
| (HIS)_2_AgBiBr_8_ | 3.1 | 252.38 | 68.9 | single crystal | out-of-plane | [S5] |
| (NH_4_)_3_Bi_2_I_9_ | 6.5 | 803 | 55 | single crystal | out-of-plane | [S6] |
| (PEA)_2_PbBr_4_ | 100 | 1303.9 | 8.8 | single crystal | out-of-plane | [S7] |
| (HIS)_2_AgSbBr_8_ | 33.3 | 223 | 84.2 | single crystal | out-of-plane | [S8] |
| (Br-PEA)_2_PbI_4_ | 80 | 1003 | 36 | single crystal | in-plane | [S9] |
| (4ABA)PbI_4_ | 25 | 572 | 7.5 | single crystal | out-of-plane | [S10] |
| (Br-BA)_2_PbBr_4_ | 150 | 531.33 | 100 | single crystal | out-of-plane | [S11] |
| (*o*-F-PEA)_2_PbI_4_ | 1250 | 1724.5 | 460 | single crystal | in-plane | [S12] |
| (BA)_2_PbI_4_ | 10 | 148 | 241 | single crystal | out-of-plane | [S13] |
| (BA)_2_PbBr_4_ | 12.5 | 544.8 | 523.3 | wafer | out-of-plane | [S14] |
| (PA)_2_PbBr_4_ | 12.5 | 827.3 | 60 | wafer | out-of-plane |  |
| (PEA)_2_PbBr_4_ | 12.5 | 3416.4 | 27 | wafer | out-of-plane |  |
| (BDA)PbI_4_ | 310 | 242 | 430 | single crystal | out-of-plane | [S15] |
| EA_4_Pb_3_Br_10_ | 60 | 2228.9 | - | wafer | in-plane | [S16] |

*PEA = phenylethylamine, HIS = histamine, 4ABA = 4-aminobenzylamine, BA = butylamine, PA = propylamine, BDA = 1,4-butanediamine, EA = ethylamine.

**Supplementary References**

[S1] S. You, P. Yu, J. Wu, Z.-K. Zhu, Q. Guan et al., Weak X-ray to visible lights detection enabled by a 2D multilayered lead iodide perovskite with iodine-substituted spacer. Adv. Sci. Weinheim Baden Wurttemberg Ger. **10**(21), e2301149 (2023). <https://doi.org/10.1002/advs.202301149>
[S2] C.-C. Fan, C.-D. Liu, B.-D. Liang, M.-L. Jin, T.-Y. Ju et al., A two-dimensional hybrid lead bromide ferroelectric semiconductor with an out-of-plane polarization. Inorg. Chem. **62**(32), 12634–12638 (2023). <https://doi.org/10.1021/acs.inorgchem.3c02057>
[S3] M. Li, H. Li, W. Li, B. Li, T. Lu et al., Oriented 2D perovskite wafers for anisotropic X-ray detection through a fast tableting strategy. Adv. Mater. **34**(8), 2108020 (2022). <https://doi.org/10.1002/adma.202108020>
[S4] F. Liao, L. Lei, X. Gan, P. Li, G. Yao, Flexible 2D perovskite film for dual-mode X-ray detection and imaging. IEEE Electron Device Lett. **45**(9), 1610–1613 (2024). <https://doi.org/10.1109/LED.2024.3424470>
[S5] Y. Zhou, L. Huang, M. Wu, F. Kuang, K. An et al., A two-dimensional lead-free double perovskite for self-powered and high performance X-ray detection. J. Mater. Chem. C **12**(44), 18048–18057 (2024). <https://doi.org/10.1039/d4tc03465a>
[S6] R. Zhuang, X. Wang, W. Ma, Y. Wu, X. Chen et al., Highly sensitive X-ray detector made of layered perovskite-like (NH_4_)_3_Bi_2_I_9_ single crystal with anisotropic response. Nat. Photonics **13**(9), 602–608 (2019). <https://doi.org/10.1038/s41566-019-0466-7>
[S7] B. Jia, D. Chu, Y. Hu, Z. Zhao, J. Pi et al., Reducing energy loss of 2D perovskite single crystal for stable and efficient X-ray detection by quenching the radioluminescence. Adv. Mater. **38**(5), e03463 (2026). <https://doi.org/10.1002/adma.202503463>
[S8] Q. Fan, H. Xu, S. You, Y. Ma, Y. Liu et al., Centimeter-sized single crystals of *Dion*-jacobson phase lead-free double perovskite for efficient X-ray detection. Small Weinheim Der Bergstrasse Ger. **19**(34), e2301594 (2023). <https://doi.org/10.1002/smll.202301594>
[S9] H. Zhong, S. You, J. Wu, Z.-K. Zhu, P. Yu et al., Multiple interlayer interactions enable highly stable X-ray detection in 2D hybrid perovskites. JACS Au **4**(6), 2393–2402 (2024). <https://doi.org/10.1021/jacsau.4c00345>
[S10] Q. Fan, Y. Ma, S. You, H. Xu, W. Guo et al., *Dion*–jacobson phase perovskite crystal assembled by *π*-conjugated aromatic spacer for X-ray detectors with an ultralow detection limit. Adv. Funct. Mater. **34**(9), 2312395 (2024). <https://doi.org/10.1002/adfm.202312395>
[S11] Q. Gu, K. Chen, X. Zhang, S. Wang, S. Wu et al., Butylammonium-based chiral 2D perovskite single crystals for efficient UV circularly polarized light differentiation and high-performance X-ray detection. ACS Appl. Mater. Interfaces **17**(11), 17127–17134 (2025). <https://doi.org/10.1021/acsami.5c01035>
[S12] B. Zhang, Z. Xu, C. Ma, H. Li, Y. Liu et al., First-principles calculation design for 2D perovskite to suppress ion migration for high-performance X-ray detection. Adv. Funct. Mater. **32**(15), 2110392 (2022). <https://doi.org/10.1002/adfm.202110392>
[S13] Yukta, J. Ghosh, M.A. Afroz, S. Alghamdi, P.J. Sellin et al., Efficient and highly stable X-ray detection and imaging using 2D (BA)_2_PbI_4_ perovskite single crystals. ACS Photonics **9**(11), 3529–3539 (2022). <https://doi.org/10.1021/acsphotonics.2c00776>
[S14] W. Wu, J. Zhang, C. Liu, J. Zhang, H. Lai et al., Spontaneous cooling enables high-quality perovskite wafers for high-sensitivity X-ray detectors with a low-detection limit. Adv. Sci. Weinheim Baden Wurttemberg Ger. **11**(46), e2410303 (2024). <https://doi.org/10.1002/advs.202410303>
[S15] Y. Shen, Y. Liu, H. Ye, Y. Zheng, Q. Wei et al., Centimeter-sized single crystal of two-dimensional halide perovskites incorporating straight-chain symmetric diammonium ion for X-ray detection. Angew. Chem. Int. Ed. **59**(35), 14896–14902 (2020). <https://doi.org/10.1002/anie.202004160>
[S16] J. Wu, Q. Yin, S. You, R. Li, C. Lin et al., Oriented perovskite ferroelectric wafers for sensitive self-driven X-ray detection. Nano Res. **19**(7), 94908302 (2026). <https://doi.org/10.26599/nr.2025.94908302>
